# Supplementary material for: Antisense Oligonucleotide in LNA-Gapmer Design Targeting TGFBR2—A Key Single Gene Target for Safe and Effective Inhibition of TGFβ Signaling
Source: Int J Mol Sci. 2020 Mar 12;21(6):1952. doi: 10.3390/ijms21061952 (PMC7139664; doi:10.3390/ijms21061952)
Supplement: Supplementary file 1 [file ijms-21-01952-s001.pdf]

Supplementary Data

Supplementary Table 1

Supplementary Table 1: BLASTn[1] query results for TGFBR2 sequences. (A) human TGFBR2 sequence is highlighted in blue and used as reference for comparison with other species (% identiy). (B) shows the TGFBR2 percent identity of selected species as a matrix.[1] https://blast.ncbi.nlm.nih.gov; [2] NCBI RefSeq release 98 (January 2020).

A

**Accession [2]**	**TGFBR2 Name**	**Query Coverage**	**Percent Identity**
Select seq NM_003242.6	Human - Homo sapiens transforming growth factor beta receptor 2 (TGFBR2), transcript variant 2, mRNA	100%	100%
Select seq XM_005545564.2	Cynomolgus monkey - PREDICTED: Macaca fascicularis transforming growth factor beta receptor II (TGFBR2), transcript variant X1, mRNA	99%	96%
Select seq XM_005634331.3	Dog - PREDICTED: Canis lupus familiaris transforming growth factor beta receptor 2 (TGFBR2), transcript variant X1, mRNA	90%	83%
Select seq XM_021071493.1	Pig - PREDICTED: Sus scrofa transforming growth factor beta receptor 2 (TGFBR2), mRNA	97%	79%
Select seq NM_001261151.1	Rhesus monkey - Macaca mulatta transforming growth factor beta receptor 2 (TGFBR2), mRNA	46%	98%
Select seq NM_009371.3	Mouse - Mus musculus transforming growth factor, beta receptor II (Tgfbr2), transcript variant 1, mRNA	79%	83%
Select seq NM_031132.3	Rat - Rattus norvegicus transforming growth factor, beta receptor 2 (Tgfbr2), mRNA	45%	84%

B

**TGFBR2 Percent Identity**	Human	Cynomolgus monkey	Dog	Pig	Rhesus monkey	Mouse	Rat
Human	100	96	83	79	98	83	84
Cynomolgus monkey	96	100	82	79	100	82	86
Dog	83	82	100	80	90	83	85
Pig	79	79	80	100	85	85	84
Rhesus monkey	98	100	90	85	100	84	86
Mouse	83	82	83	85	84	100	93
Rat	84	86	85	84	86	93	100

Supplementary Table 2

Supplementary Table 2: NVP-13 ASO sequence specificity. List of sequence comparison with up to 5 false base pairs allowed in all transcripts of human TGFBR2, BMPR2 and Activin receptor 2 versus ASO X07091 (NVP-13). NVP-13 has 0 mismatches in all human TGFBR2 transcripts, 4 mismatches for human BMPR2 transcripts and more then 5 mismatches for ACVR2B (therefore not listed). [1]NCBI RefSeq release 98 (January 2020).

ID	Oligo length	Accession [1]	Gene ID	Gene symbol	Description	On-target site (5'-3')	Off-target site (5'-3')	Compound sequence (5'-3')	Number of mismatches
X07091	16	NM_001204.7	659	BMPR2	Homo sapiens bone morphogenetic protein receptor type 2 (BMPR2), mRNA	UACUGGUC CAUUCAUG	UACUAUUU CAUUCAAG	CATGAATG GACCAGTA	4
X07091	16	XM_011511687.1	659	BMPR2	PREDICTED: Homo sapiens bone morphogenetic protein receptor type 2 (BMPR2), transcript variant X1, mRNA	UACUGGUC CAUUCAUG	UACUAUUU CAUUCAAG	CATGAATG GACCAGTA	4
X07091	16	NM_001024847.2	7048	TGFBR2	Homo sapiens transforming growth factor beta receptor 2 (TGFBR2), transcript variant 1, mRNA	UACUGGUC CAUUCAUG	UACUGGUC CAUUCAUG	CATGAATG GACCAGTA	0
X07091	16	NM_003242.6	7048	TGFBR2	Homo sapiens transforming growth factor beta receptor 2 (TGFBR2), transcript variant 2, mRNA	UACUGGUC CAUUCAUG	UACUGGUC CAUUCAUG	CATGAATG GACCAGTA	0
X07091	16	XM_011534043.2	7048	TGFBR2	PREDICTED: Homo sapiens transforming growth factor beta receptor 2 (TGFBR2), transcript variant X1, mRNA	UACUGGUC CAUUCAUG	UACUGGUC CAUUCAUG	CATGAATG GACCAGTA	0
X07091	16	XM_011534045.3	7048	TGFBR2	PREDICTED: Homo sapiens transforming growth factor beta receptor 2 (TGFBR2), transcript variant X2, mRNA	UACUGGUC CAUUCAUG	UACUGGUC CAUUCAUG	CATGAATG GACCAGTA	0
X07091	16	XM_017007106.1	7048	TGFBR2	PREDICTED: Homo sapiens transforming growth factor beta receptor 2 (TGFBR2), transcript variant X3, mRNA	UACUGGUC CAUUCAUG	UACUGGUC CAUUCAUG	CATGAATG GACCAGTA	0

Supplementary Figure 1

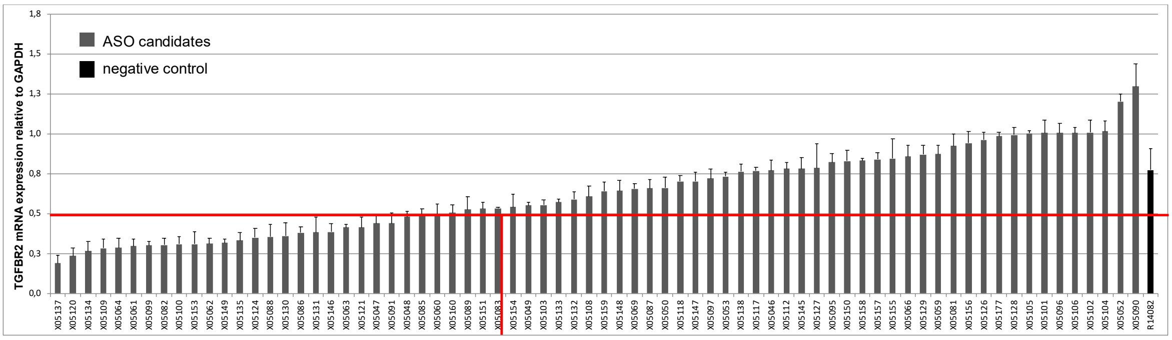

Supplementary Figure 1: Transfection experiments at low dose (5nM). After high dose (20nM) transfection experiments with lipofectamine 2000 72 of 110 potential candidates remain for low dose (5nM) lipofectamine 2000 transfection experiments (A549 cells). Remaining TGFBR2 mRNA after 24 hours of incubation with different antisense oligonucleotides (ASO) as measured in a bDNA assay (n = 4) relative to the reference gene GAPDH. Thus, low dose experiments of first screening round revealed 30 active candidates reducing TGFBR2 mRNA by approximately 50 % or more (X05137 – X05083). These 30 candidates were further tested in direct incubation experiments (gymnotic delivery). TGFBR2 expression was normalized to respective PBS-treated control cells. Mean values of n = 4 with SD are shown. XO-number = ASOs, R14082 ASO as negative control directed against Aha-4 with readout for remaining TGFBR2 mRNA.

Supplementary Figure 2

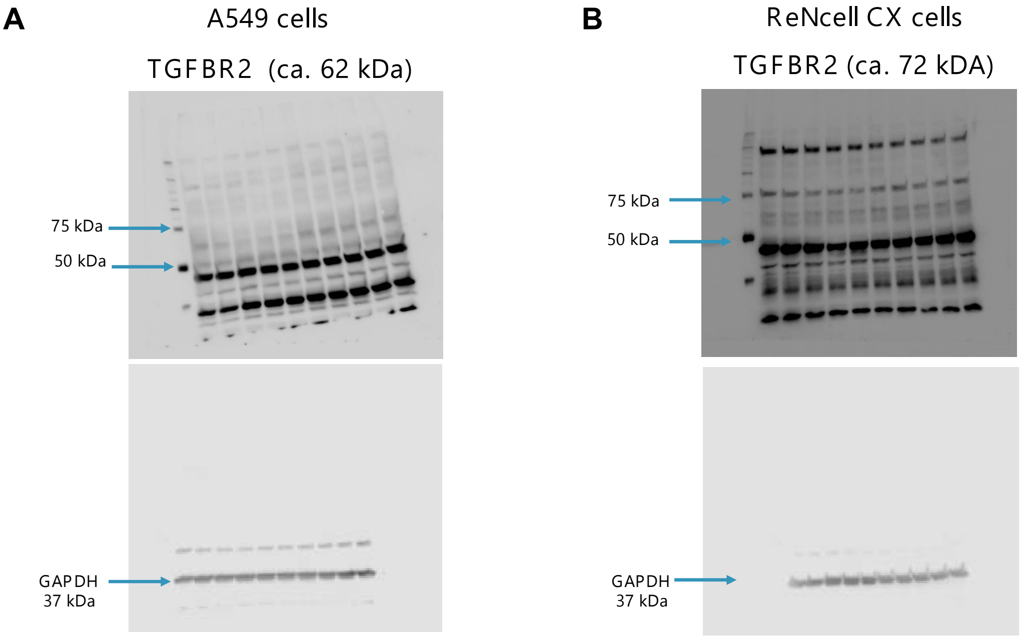

C

Lane	1	2	3	4	5	6	7	8	9	10	11
	Precision Plus Protein™ Ladder (BioRad 161-0376)	12d Untreated	1d X07091 = NVP13	4d X07091 = NVP13	8d X07091 = NVP13	12d X07091 = NVP13	12d Untreated	1d Control Oligo	4d Control Oligo	8d Control Oligo	12d Control Oligo

Supplementary Figure 2: Original Western Blot. Original Images of Western Blot (WB) against TGFBR2 (Biorybt) and Housekeeper protein GAPDH (cell signaling) in A549 (A) and ReNcell CX® cells (B). All values were normalized to untreated controls and analysis of WB membranes was done by Image studio lite software. (C) SDS-acrylamide gel loading scheme for samples in (A) and (B).

Supplementary Figure 3

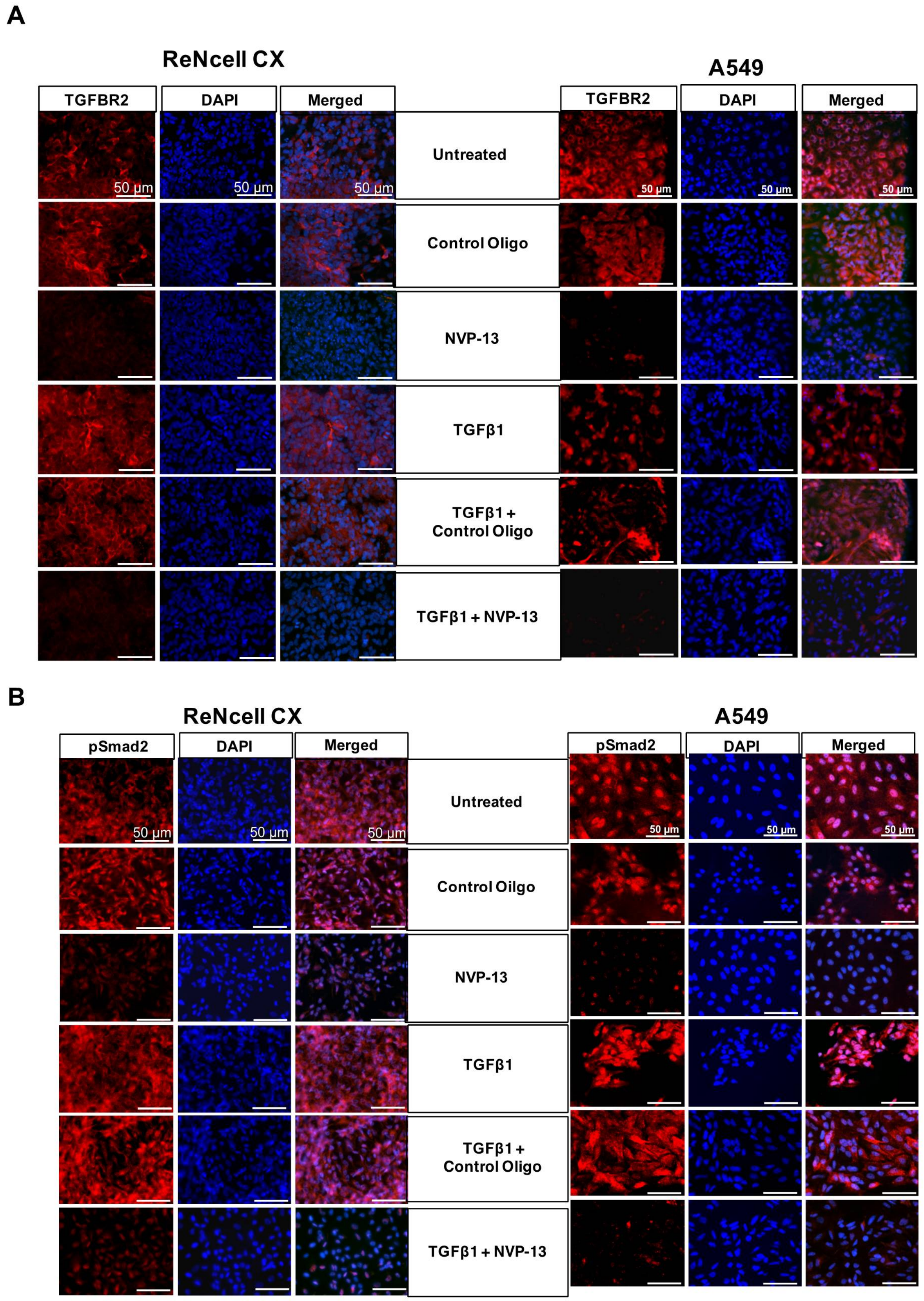

C

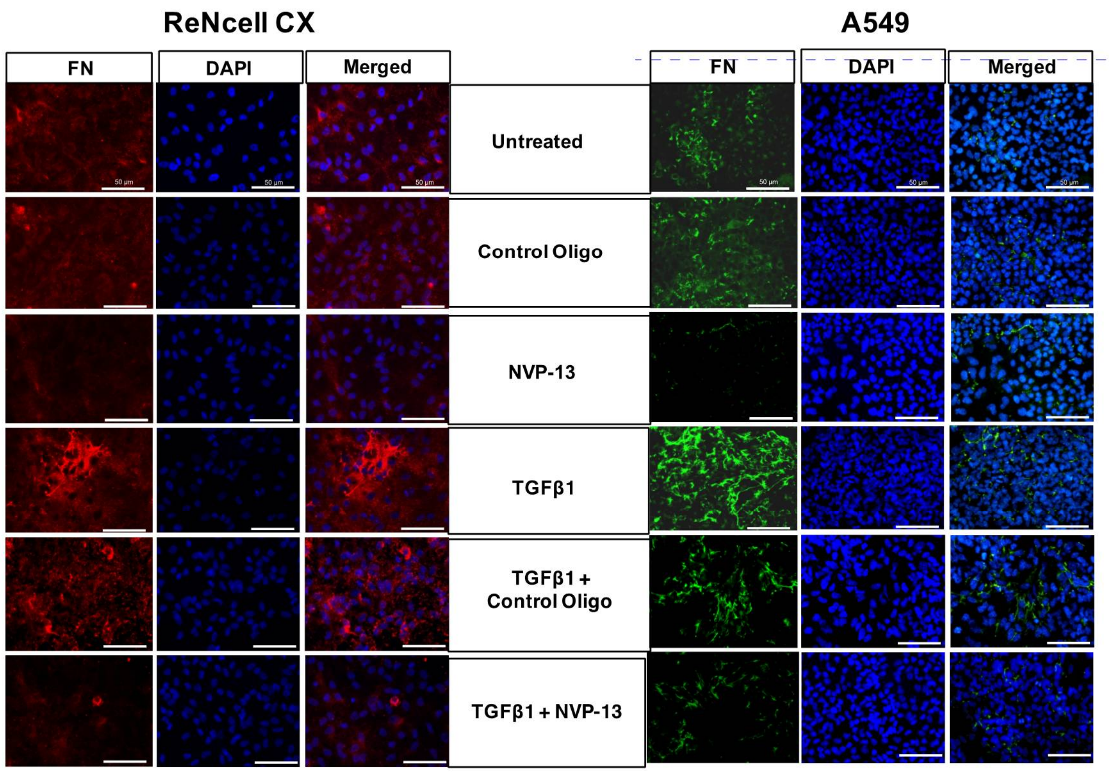

Supplementary Figure 3: Immunocytochemical stainings against TGFBR2, pSmad2. (A) shows labeling with an antibody against TGFBR2 (Millipore #06-227, left column; red) in ReNcell CX (left) and A549 (right). Gymnotic transfer of NVP-13 showed an efficient downregulation of target TGFBR2 for both cell lines. (B) Inhibition of TGFβ-signaling was verified by labeling with an antibody against pSmad2 (cell signaling cs3104s, left column; red) in ReNcell CX (left) and A549 (right). (C) Functional inhibition of TGFβ-signaling mediated by gymnotic transfer of NVP-13 was confirmed by labeling with an antibody against FN (Proteintech #15613-1-AP, left column) in ReNcell CX (left: red) and A549 (right: green). Nuclear DNA was stained with DAPI (central column, blue). Labeling with antibodies in ReNcell CX (left) and A549 (right) was performed after TGFβ1-preincubation (ReNcell CX: 4 d, A549: 48 h) and subsequently gymnotic transfer of NVP-13 for 8 d (ReNcell CX) or 72 h (A549). Nuclear DNA was stained with DAPI (central column, blue). Examination of cells was performed by fluorescence microscopy (Zeiss, Zeiss Axio® Observer.Z1). Images were analyzed with Image J Software and CorelDRAW® X7 Software. N = 3.

Supplementary Figure 4
A

		Purity IP-RP-HPLC-ESI/MS	
		Rel. Area in %	
**Stock Solution (1 mg/ml)**	**Time Point (months)**	**Rel. Area NVP-13 %**	**OOS-Level +/- of relative area from purity T=0**
NVP-13 Stock 1	0	89.25	0.0 %
NVP-13 Stock 2		89.11	0.0 %
NVP-13 Stock 1	1 month	89.82	0.6 %
NVP-13 Stock 2		87.45	1.9 %
NVP-13 Stock 1	3 month	89.51	0.3 %
NVP-13 Stock 2		89.62	0.6 %
NVP-13 Stock 1	6 months	89.10	0.2 %
NVP-13 Stock 2		88.98	0.1 %
NVP-13 Stock 1	6 months (reanalysis batch)	89.16	0.1 %
NVP-13 Stock 2		89.19	0.1 %
NVP-13 Stock 1	12 months	89.09	0.2 %
NVP-13 Stock 2		89.39	0.3 %

B

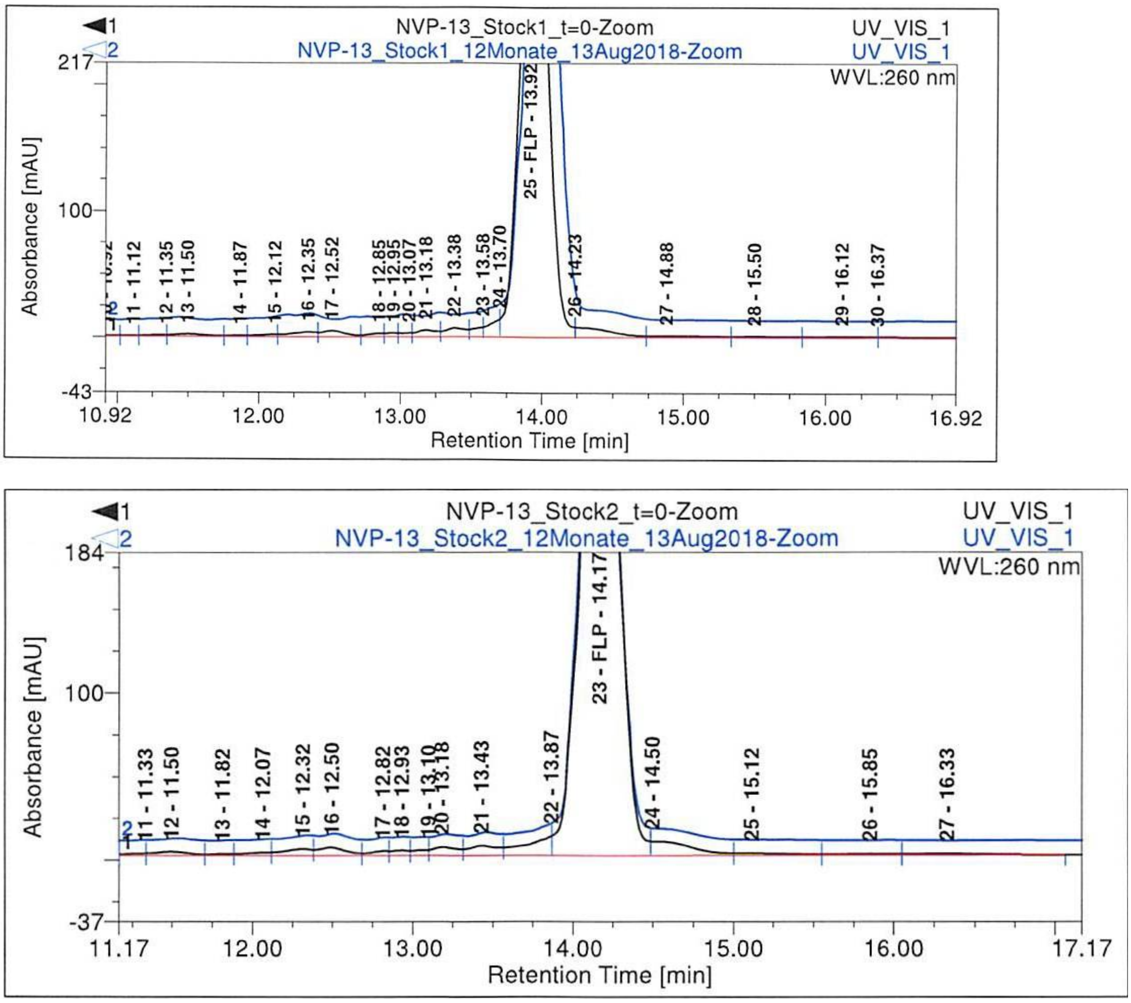

Supplementary Figure 4: Integrity of NVP-13 at -20°C up to 12 months. Long-term stability was assessed applying a denaturizing Ion-Pair-Reversed-Pair High Performance Liquid Chromatography (IP-RP-HPLC) with Electrospray-Ionization (ESI) / Mass Spectrometry (MS) for determination of relative purity and identity of NVP-13. (A) Aliquots of NVP-13 with a stock solution of approximately 1 mg/ml in 0.9 % NaCl (saline) were stored at -20°C +/- 5°C for 0 (n = 2), 1 (n = 2), 3 (n = 2), 6 (n = 4) and 12 (n = 2) months. The change of relative purity of freshly dissolved aliquot should be $\leq$10 %. Thus, all tested aliquots for NVP-13 was stable at -20°C for up to 12 months. Values are shown as mean, OOS = Out-of-Specification (B) Anion-Exchange High Performance Liquid Chromatography (AEX-HPLC) chromatograms for tested stock1 and 2. NVP-13 concentration was 1 mg/ml. Chromatograms for T = 0 analyses are shown in black. Chromatograms for T =12 months analyses are shown in blue. No differences in chromatogram overlay were detected. Results confirmed that NVP-13 in saline was stable at least for 12 months at -20°C.

Supplementary Figure 5

A

Stability at 5°C +/- 3°C up to 1 week		
**Stock Solution**	**Time Point (week)**	**Purity IP-RP-HPLC-UV/MS Rel. Area NVP-13 (%)**
NVP-13 0.5 mg/ml	0	89.8
NVP-13 0.57 mg/ml	1	90.4
NVP-13 5.70 mg/ml		91.0

B

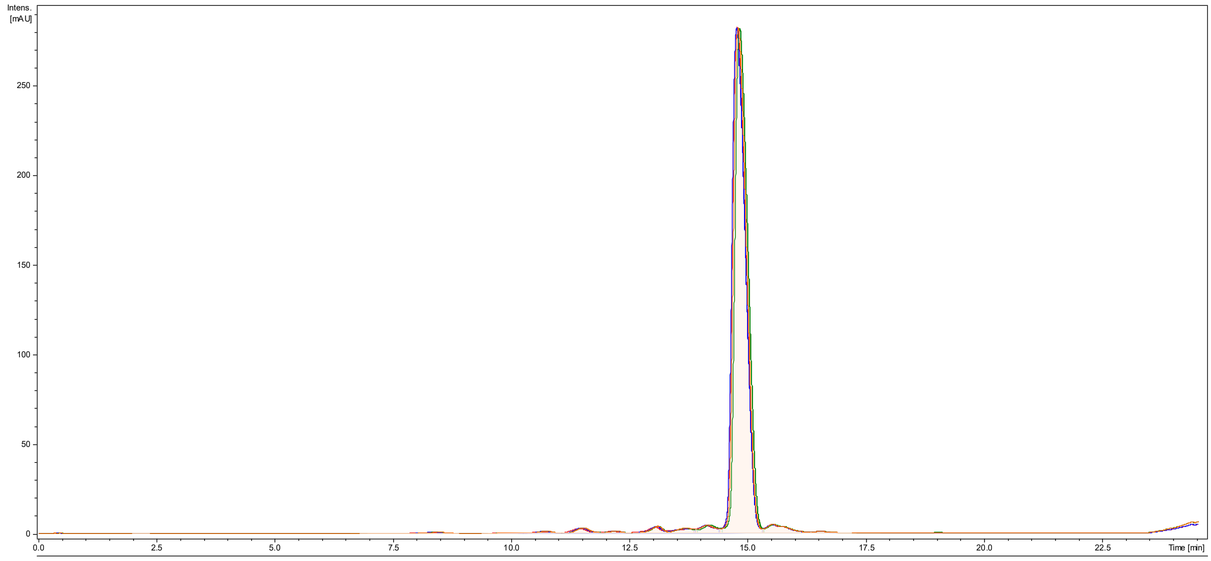

Supplementary Figure 5: Integrity of NVP-13 at 5°C tested for 1 week. Integrity was assessed applying a denaturizing Ion-Pair-Reversed-Pair High Performance Liquid Chromatography (IP-RP-HPLC) with UV / Mass Spectrometry (MS) for determination of identity of NVP-13. (A) Aliquots of NVP-13 with a stock solution of 0.57 and 5.7 mg/ml in 0.9 % NaCl (saline) were stored at 5°C +/- 3°C for 0 (n = 1) and 1 week (n = 1 of each concentration). The HPLC purity ranged from 90.4 % to 91.0 %. Freshly tested NVP-13 stock solution showed HPLC purity of 89.8 %. Discrepancy for purity/integrity are within the method variability. (B) In an overlay of all chromatograms it is shown that the general peak profile is comparable for all samples. Results confirmed that NVP-13 in saline was stable for at least 1 week at 5°C.

Supplementary Figure 6

A

		Purity IP-RP-UPLC-UV/ESI/MS			
		Rel. Area in %			
**Stock Solution**	**Time Point (month)**	**Full length product**	**3'+ 5' (N-2)**	**3' (N-1)**	**5' (N-1)**
NVP-13 0.25 mg/ml	0	95.3	0.6	0.8	0.9
NVP-13 0.5 mg/ml	Spiked 0	83.7	4.5	3.3	6.1
NVP-13 0.25 mg/ml	1 month	92.4	0.6	0.8	0.9
NVP-13 0.5 mg/ml	Spiked 1 month	81.2	4.5	3.1	6.3

B

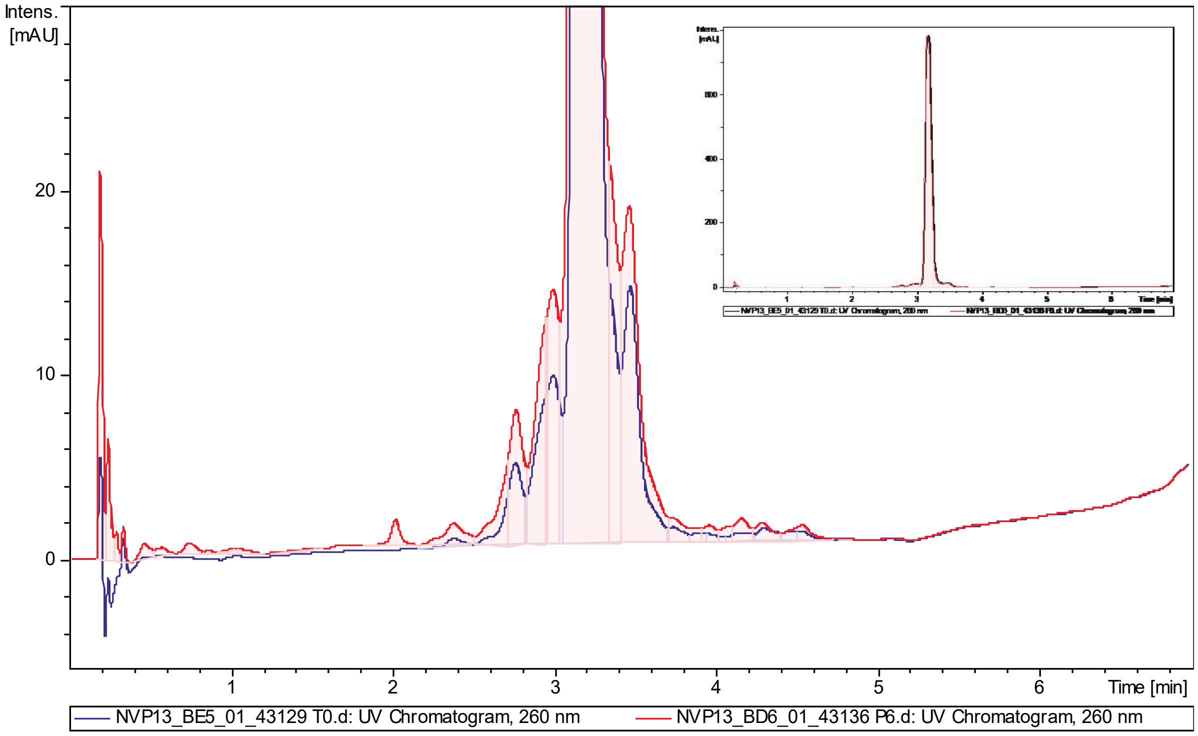

Supplementary Figure 6: Integrity of NVP-13 at 37°C tested for up to 1 month. Integrity was assessed applying a denaturizing Ion-Pair-Reversed-Pair Ultra-High-Performance Liquid Chromatography (IP-RP-UPLC) with UV / ESI-Mass Spectrometry (MS) for determination of identity of NVP-13. (A) Aliquots were tested for T= 0 (n =1) and 1 month (n =1). Aliquots of both time points were analyzed for full-length product (FLP). Separate samples were spiked with each of three impurities (3' (N-1), 5' (N-1), 3' + 5' (N-2)), in order to calibrate for identification these impurities. No changes in purity or in the relative composition of the reference impurities over 1 month was observed. (B) Overlays of T=0 (blue curves) and T= 1 month (red curve) IPRP-UPLC chromatograms for NVP-13. Inset confirm exact overlap of FLP peak (main peak) (ordinate axis, 800 mAU full-scale); main panel chromatograms are shown with ordinate axes at 30 mAU full-scale in order to highlight details inside peaks. Results confirmed that NVP-13 in saline was stable for at least 1 month at 37°C.
